# 

**Published:** 1878-09

**Authors:** 


### CONTENTS.

### COMMUNICATIONS.

Diagnosis. *By L. C. Ingersoll*........................................ 353

Embryology. *By Dr. W. H. Atkinson*........................................ 367

Therapeutical Treatment of Dental Pulps. *By Dr. A. F. McLain*... 376

### EDITORIAL.

"Bleaching Teeth"........................................ 386

The American Dental Association........................................ 394

Disease of the Gums........................................ 396

Transactions........................................ 396

